# Repurposing Avasimibe to Inhibit Bacterial Glycosyltransferases

**DOI:** 10.3390/pathogens11030370

**Published:** 2022-03-17

**Authors:** Md Kamrul Hasan, Samir El Qaidi, Peter McDonald, Anuradha Roy, Philip R. Hardwidge

**Affiliations:** 1 College of Veterinary Medicine, Kansas State University, Manhattan, KS 66506, USA; kamrulbd@ksu.edu (M.K.H.); elqaidi@vet.k-state.edu (S.E.Q.); 2 Infectious Diseases Assay Development/HTS Laboratory, University of Kansas, Lawrence, KS 66047, USA; petemcd@ku.edu (P.M.); anuroy@ku.edu (A.R.)

**Keywords:** type three secretion system effectors, glycosyltransferase, enteric bacteria

## Abstract

We are interested in identifying and characterizing small molecule inhibitors of bacterial virulence factors for their potential use as anti-virulence inhibitors. We identified from high-throughput screening assays a potential activity for avasimibe, a previously characterized acyl-coenzyme A: cholesterol acyltransferase inhibitor, in inhibiting the NleB and SseK arginine glycosyltransferases from *Escherichia* *coli* and *Salmonella enterica*, respectively. Avasimibe inhibited the activity of the *Citrobacter rodentium* NleB, *E. coli* NleB1, and *S. enterica* SseK1 enzymes, without affecting the activity of the human serine/threonine N-acetylglucosamine (O-GlcNAc) transferase. Avasimibe was not toxic to mammalian cells at up to 200 µM and was neither bacteriostatic nor bactericidal at concentrations of up to 125 µM. Doses of 10 µM avasimibe were sufficient to reduce *S. enterica* abundance in RAW264.7 macrophage-like cells, and intraperitoneal injection of avasimibe significantly reduced *C. rodentium* survival in mice, regardless of whether the avasimibe was administered pre- or post-infection. We propose that avasimibe or related derivates created using synthetic chemistry may have utility in preventing or treating bacterial infections by inhibiting arginine glycosyltransferases that are important to virulence.

## 1. Introduction

We have characterized a conserved group of type III secretion system (T3SS) effector proteins that inhibit innate immune responses to infection [1,2,3,4,5]. These proteins (named NleB in enterohemorrhagic and enteropathogenic *Escherichia coli* (EHEC and EPEC) and SseK in *Salmonella enterica*) are glycosyltransferases that are important for bacterial virulence. These enzymes glycosylate host protein substrates with β-*D*-N-acetylglucosamine (GlcNAc) on arginine residues. 

Several “death domain”-containing proteins such as the Fas-associated protein with death domain (FADD), tumor necrosis factor receptor type 1-associated death domain protein (TRADD), and RIPK1 are substrates of the NleB/SseK glycosyltransferases [6]. NleB1 disrupts tumor necrosis factor receptor (TNFR)-associated factor (TRAF) signaling, leading to inhibition of the proinflammatory NF-κB pathway [7]. Another target of NleB1 is glyceraldehyde 3-phosphate dehydrogenase (GAPDH). In addition to its role in glycolysis, GAPDH binds to TRAF proteins and stimulates TRAF polyubiquitination [4].

Arginine glycosylation is biologically important because the glycosylation of arginines on host protein substrates leads to their irreversible inactivation and disrupts the normal functioning of the innate immune response. Mammals do not have the enzymatic machinery to add GlcNAc residues to arginine (N-GlcNAc), while this modification is absolutely critical for *E. coli* and *Salmonella* virulence. Inhibitors that prevent the formation of this unusual post-translational modification represent a potentially novel way to combat these infections. Non-traditional antibacterial therapeutic strategies have recently been reviewed and are of emerging interest [8].

**Figure 1:**
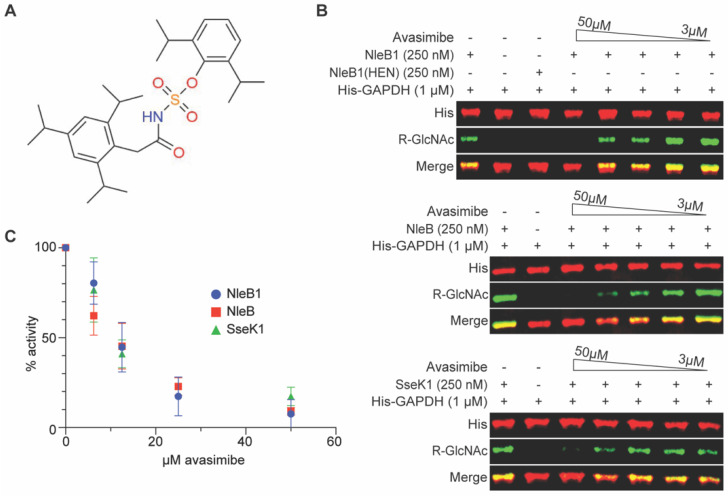
In vitro glycosylation assays. (**A**) Avasimibe structure. (**B**) Western blot results for avasimibe inhibition of NleB1, NleB, and SseK1 (250 nM) glycosylation of glyceraldehyde 3-phosphate dehydrogenase (GAPDH, 1 µM). Avasimibe was added at concentrations from 3 to 50 µM. The NleB1(HEN) enzyme is an inactive negative control [5]. (**C**) Quantification of Western blot signal intensities from replicates of in vitro glycosylation assays, *n* = 3 independent experimental replicates.

We previously developed a high-throughput screening (HTS) assay to identify NleB/SseK inhibitors [9,10]. Here, we show that avasimibe (Figure 1A), an acyl-coenzyme A: cholesterol acyltransferase inhibitor [11], also inhibits NleB and SseK enzyme activity, leading to reduced pathogen colonization in macrophage and mouse models of *Salmonella* and *Citrobacter rodentium* infection, respectively.

## 2. Results

### 2.1. Avasimibe Inhibits NleB1 and Its Orthologs

We previously published the results of HTS assays in which we identified several small molecules (100066N, 102644N, and YM155) capable of inhibiting NleB/SseK enzyme activity in vitro [9,10]. In those previous studies, we also discovered a potential activity for avasimibe in inhibiting EHEC NleB1 activity. We first validated the HTS data by quantifying the extent to which avasimibe could prevent Arg-glycosylation of the GAPDH substrate by the EHEC NleB1, *Citrobacter* NleB, and *Salmonella* SseK1 enzymes in vitro. We conducted the glycosylation assays in the presence of 2-fold serial dilutions of avasimibe. Avasimibe inhibited each enzyme in a concentration-dependent manner, with apparent IC_50s_ of ~10 µM (Figure 1B,C). 

### 2.2. Avasimibe Does Not Inihibit the Mammalian OGT Enzyme, Is Not Toxic to Mammalian Cells, and Is Neither Bacteriostatic nor Bacteriocidal

**Figure 2:**
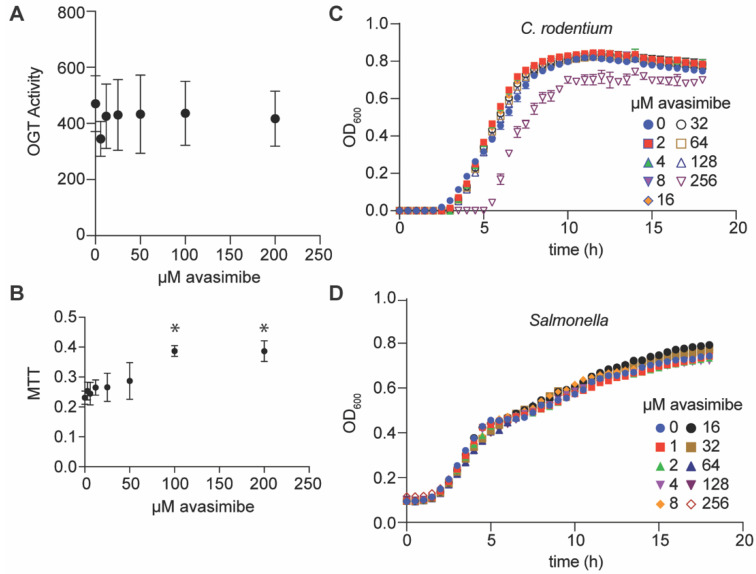
OGT activity, cytotoxicity, and bacterial growth assays. (**A**) OGT activity assay. OGT activity was measured by using a UDP-Glo assay in the presence of avasimibe, n = 3 independent experimental replicates. (**B**) Cell cytotoxicity as measured by performing 3-(4,5-dimethylthiazol-2-yl)-2,5-diphenyltetrazolium bromide (MTT) assays. Avasimibe was added to RAW264.7 cells for 24 h, and cell viability was assayed by monitoring MTT signal intensity, n = 3 independent experimental replicates. Asterisks (*) indicate significantly different MTT signals as compared to untreated cells, *p* < 0.05, Dunn’s multiple comparisons test. (**C**,**D**) Bacterial growth assays. *C. rodentium* and *S. enterica* were cultured in LB media at 37 °C in the presence of the indicated concentrations of avasimibe. Bacterial growth was monitored as a function of time, *n* = 3 independent experimental replicates.

We next examined whether avasimibe affected the activity of the human serine/threonine N-acetylglucosamine (O-GlcNAc) transferase (OGT) that regulates protein glycosylation [12]. To assess OGT activity as a function of avasimibe concentration, we used a bioluminescence-based UDP-Glo glycosyltransferase assay. Avasimbe had no effect on human OGT activity in vitro at concentrations of up to 200 µM (Figure 2A). Avasimibe was also found to be nontoxic to mammalian cells at concentrations of up to 50 µM, as determined by conducting a 3-(4,5 dimethylthiazol-2-yl)-2,5-diphenyltetrazolium bromide (MTT) assay (Figure 2B). Avasimbe had no effect on the growth rates of *C. rodentium* or *Salmonella enterica* at concentrations of up to 125 µM when these bacteria were grown in LB broth (Figure 2C,D). Thus, avasimibe does not appear to act as a general bacteriostatic or bactericidal agent. 

### 2.3. Avasimibe Inhibits Salmonella and C. rodentium Survival In Vivo

**Figure 3:**
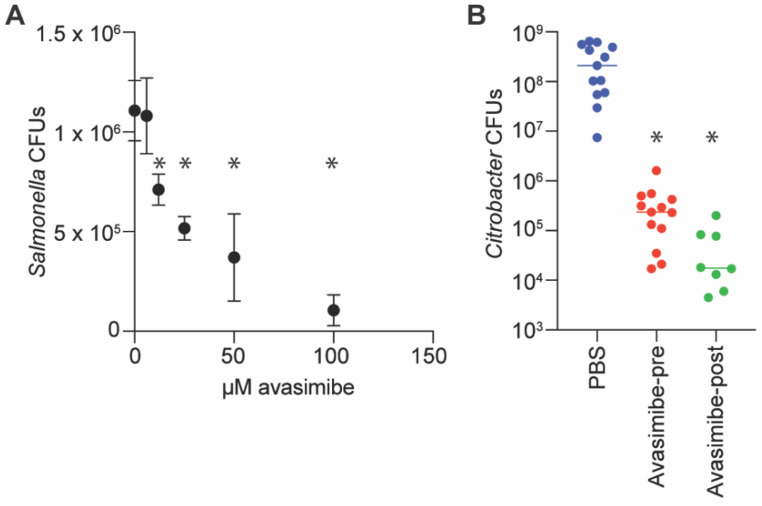
Infection assays. (**A**) *Salmonella* infection assays. RAW264.7 cells were seeded at 1 × 10^5^ cells/well in 24-well plates, and avasimibe was added 1 h before infection with 10^6^ CFUs of *Salmonella* for 30 min. Cells were incubated in medium containing 100 µg/mL gentamicin for 1 h, and then in 10 µg/mL gentamicin for an additional 23 h. Bacteria were released from RAW264.7 cells using 1% saponin, diluted in PBS, and plated for colony counts, *n* = 3 independent experimental replicates. Asterisks (*) indicate significantly different *Salmonella* CFUs as compared to untreated macrophages, *p* < 0.05, Dunn’s multiple comparisons test. (**B**) *C. rodentium* infections of mice. Mice were infected via oral gavage with 10^9^ CFUs of *C. rodentium* in 100 µL PBS. Avasimibe (25 mg/kg) was administered to one group of mice via intraperitoneal (IP) injection immediately before oral gavage. Avasimibe (5 mg/kg) was provided to another group of mice via IP injection at 24 and 48 h after oral gavage. Asterisks indicate significantly different CFUs as compared to untreated mice, *p* < 0.05, Dunn’s, *n* = 8–13 mice.

We quantified the impact of avasimibe on *Salmonella* survival in cell culture to determine whether it can be used to reduce pathogen loads in mammalian cells. When avasimibe was provided to RAW264.7 cells prior to *Salmonella* infection at concentrations greater than 10 µM, the amount of intracellular *Salmonella* was significantly reduced 24 h later (Figure 3A). 

We performed a mouse infection experiment to determine whether avasimibe inhibition of NleB had any effect in vivo. We administered avasimibe via intraperitoneal injection at doses of either 25 mg/kg immediately prior to infecting mice with *C. rodentium*, or 5 mg/kg at 24 and 48 h post-infection. After 7 days, we euthanized the mice and quantified the amount of *C. rodentium* in the colon. We observed a significant reduction in *C. rodentium* in mice treated with avasimibe, as compared to untreated mice, regardless of whether the avasimibe was administered pre- or post-infection (Figure 3B). These findings suggest that avasimibe could be used as an anti-virulence small molecule.

## 3. Discussion

We discovered here that avasimibe, a previously characterized ACAT inhibitor, also inhibits the NleB and SseK arginine glycosyltransferases. Avasimibe has a well-documented solubility and safety profile [13], although it caused a potential reduction in the potency of Lipitor [11] and was thus not ultimately used to treat hyperlipidemia or atherosclerosis. Avasimibe also suppresses tumor proliferation and metastasis via the E2F-1 signaling pathway and has potential utility in treating prostate cancer [14]. Avasimibe alleviates insulin resistance in diet-induced obese mice [15]. Avasimbe impedes tick embryo development by interfering with tick lipid metabolism, making ticks more susceptible to bacterial infection [16]. Avasimibe has also shown encouraging results in inhibiting glioma cell proliferation [17]. Additionally, avasimibe was identified as a potential hepatitis C virus inhibitor, where it targets the assembly of infectious viral particles [18]. 

Our glycosylation assay results suggest that avasimibe has an IC_50_ of ~10 µM against the NleB/SseK enzymes. At these concentrations, avasimibe has no substantial inhibitory effect on pathogen growth, no cytotoxicity to mammalian cells, and does not inhibit the human OGT enzyme. Finally, our in vivo findings suggest that avasimibe is well tolerated in mice and significantly reduces *C. rodentium* loads in the intestine, regardless of whether it is administered pre- or post-infection. 

Numerous previous investigations have established the safety profile of avasimibe in mice. These studies included prolonged administration of similar doses of avasimibe as those used in our studies, with no reported adverse effects. For example, in a study of Lewis lung carcinoma in mice, 15 mg/kg of avasimibe was administered for 25 days via IP injection with no adverse effects in mice [19]. A similar study used a 7.5 mg/kg dose of avasimibe via IP injection for 30 days and found no adverse effects [20]. A 10 mg/kg dose of avasimibe for up to 22 weeks of daily administration had no effect on body weight or food intake of mice [21].

We have now identified and studied a number of inhibitors that have the potential to function as anti-virulence therapeutics against enteric pathogens. We were indeed surprised to find that avasimibe functions as an inhibitor of bacterial glycosyltransferases. Our future goals are to acquire structural information using NMR and crystallography to characterize the avasimibe binding site and mode of action. Our ultimate goal is to combine our understanding of the molecular mechanisms of action of these inhibitors to create new compounds with improved solubility, lower production costs, and good safety profiles. For example, using synthetic chemistry to modify avasimibe and reduce its interaction with the liver enzyme CYP3A4 [22] could reduce its side effects in humans while maintaining its safety and efficacy profile.

## 4. Materials and Methods

### 4.1. Cell Lines

Abelson-murine-leukemia-virus-induced, macrophage-like cells from BALB/c mice (RAW264.7) were purchased from ATCC and grown in Dulbecco’s Modified Eagle Medium (DMEM), supplemented with 10% fetal bovine serum (FBS) (Atlanta Biologicals, Minneapolis, MN, USA) and 100 µg/mL penicillin/streptomycin (Sigma, St. Louis, MO, USA) in 5% CO_2_.

### 4.2. Protein Purification

Protein purification was conducted as previously described [1]. *E. coli* BL21 (DE3) strains were cultured in LB at 37 °C until an OD600 of 0.4, after which 0.5 mM IPTG was added for 4 h. The pellet was resuspended in 50 mM sodium phosphate buffer pH 8.0 and 0.5 mg/mL lysozyme after centrifugation. After 30 min on ice with periodic shaking, the suspension was treated with 50 mM sodium phosphate buffer pH 8.0, 2 M NaCl, 8 mM imidazole, 20% glycerol, and 2% Triton X-100 for 30 min. After sonicating and centrifuging the cell lysates, the supernatant was added to 2 mL Ni-NTA resin (Qiagen, Germantown, MD, USA) for 1 h of rotation at 4 °C. The mixture was added to a Poly-Prep Chromatography Column (Bio-Rad, Hercules, CA, USA) and washed with 10 mL of 50 mM sodium phosphate buffer pH 8.0, 600 mM NaCl, 60 mM imidazole, and 10% glycerol. Proteins were eluted in 2 mL 50 mM sodium phosphate buffer pH 8.0, 600 mM NaCl, 250 mM imidazole, and 10% glycerol, then dialyzed into the same buffer lacking imidazole.

### 4.3. Glycosylation Assays

In vitro glycosylation assays were conducted as previously described [1]. Enzymes (200 nM of NleB1, NleB, or SseK1) were incubated in 50 mM Tris-HCl buffer pH 7.4, 1 mM UDP-GlcNAc, 10 mM MnCl_2_, and 1 mM DTT with 1 mM GAPDH in the presence or absence of serial dilutions of avasimibe. After a 2 h incubation at room temperature, samples were blotted with anti-R-GlcNAc and anti-His tag monoclonal antibodies (Abcam, Cambridge, MA, USA). LI-COR Image Studio software (LI-COR Biosciences, Lincoln, NY, USA) was used to measure signal intensities, and inhibition was estimated by measuring the relative reduction in substrate glycosylation.

### 4.4. OGT Assays

The UDP-GloTM Glycosyltransferase Assay Kit was used as specified by the manufacturer (Promega). OGT (200 nM) in 25 mM Tris-HCL buffer pH 7.5, 12.5 mM MgCl_2_, 0.06 mg/mL BSA, 1 mM DTT, 50 µM OGT peptide substrate, and 100 µM UDP-GlcNAc were used in the reactions, which also included avasimibe at concentrations ranging from 6 to 200 µM. The reactions were incubated at 22 °C for 1 h, and the luminescence signals were measured by using a FLUO star microplate reader (BMG Labtech, Cary, NC, USA).

### 4.5. MTT Assays

MTT tests utilizing RAW264.7 cells in the presence of 2-fold serial dilutions of avasimibe from 3 to 200 µM concentration were performed as specified by Millipore. Formazan absorbance was measured at 570 nm using a BioTek Microplate reader (BioTek, Winooski, VT, USA).

### 4.6. Bacterial Growth Assays

Bacterial cultures were grown overnight, diluted 1:200 in LB, and then grown at 37 °C for 18 h in the presence of 2-fold serial dilutions of avasimibe (0–256 µM). The absorbance of the culture medium at OD_600_ was used to monitor bacterial growth.

### 4.7. Macrophage Infections

RAW264.7 cells were seeded at 1 × 10^5^ cells/well in TCP 24-well tissue culture plates, and avasimibe (6–100 µM) was added 1 h before infection. *Salmonella* cultures were grown overnight, and 10^6^ CFUs were added to each well for 30 min. Cells were treated with 100 µg/mL gentamicin for 1 h and then with 10 µg/mL gentamicin for an additional 23 h. Bacteria were released from RAW264.7 cells using 1% saponin (Sigma), diluted in PBS, and plated to enumerate the number of intracellular *Salmonella*. 

### 4.8. Mouse Infections

Five-week-old C57BL/6 mice (Jackson Laboratory) were housed at Kansas State University. *C. rodentium* DBS100 was cultivated in LB broth with shaking at 200 rpm at 37 °C overnight. Mice were infected via oral gavage with 10^9^ CFUs of *C. rodentium* in 100 µL PBS. Avasimibe was administered to one group of mice via intraperitoneal (IP) injection immediately before oral gavage. Avasimibe was provided to another group of mice via IP injection at 24 and 48 h after oral gavage. Mice were euthanized 7 days after infection, colons were homogenized, serially diluted, and plated on MacConkey agar. The following day, colonies were enumerated and plotted to compare *C. rodentium* loads between experimental groups.
